# Bacterial etiology and antibiotic susceptibility pattern of diabetic foot infections in Tabriz, Iran

**DOI:** 10.3205/dgkh000245

**Published:** 2015-02-02

**Authors:** Mohammad Taghi Akhi, Reza Ghotaslou, Mohammad Asgharzadeh, Mojtaba Varshochi, Tahereh Pirzadeh, Mohammad Yousef Memar, Abed Zahedi Bialvaei, Hasan Seifi Yarijan Sofla, Naser Alizadeh

**Affiliations:** 1 Research Center of Infectious and Tropical Disease, Tabriz University of Medical Sciences, Tabriz, Iran; 2 Department of Bacteriology and Virology, School of Medicine, Tabriz University of Medical Sciences, Iran; 3 Paramedical Faculty, Tabriz University of Medical Sciences, Tabriz, Iran

**Keywords:** diabetic foot infections, antibiotic susceptibility pattern, anaerobic bacteria

## Abstract

**Aim: **The aim of this study was to investigate anaerobic and aerobic bacteria profile and determination of antibiotic susceptibility pattern in aerobic bacteria.

**Method:** Specimens were cultured using optimal aerobic and anaerobic microbiological techniques. Identification of bacterial isolates was performed by standard microbiological methods and antibiotic susceptibility testing was performed according to the guidelines of Clinical and Laboratory Standards Institute (CLSI).

**Result:** 92 bacterial strains were isolated from 60 samples of diabetic foot ulcers. Predominant aerobic bacteria isolated from these infections were *S. aureus* (28%) followed by Enterobacteriaceae family (24%) including *Escherichia coli* (15%), *Citrobacter *spp. (4%), *Enterobacter *spp. (4%), and coagulase-negative *Staphylococcus *spp. (17%),* Enterococcus *spp. (15%), *Pseudomonas aeruginosa* (7%) and *Acinetobacter *spp. (4%). No *Clostridium *spp. were isolated and 4% *Bacteroides fragilis* obtained from anaerobic culture. All Gram-positive isolates were susceptible to linezolid while all Enterobacteriaceae showed sensitivity to imipenem.

**Conclusion:** Most of DFIs specimens were poly microbial infection and predominant bacteria were *S. aureus* and *B. fragilis*. These wounds may require use of combined antimicrobial therapy for initial management.

## Zusammenfassung

**Zielsetzung:** Es sollte die bakterielle Ätiologie (anaerobe und aerobe Flora) und Antibiotikaempfindlichkeit von Erregern beim diabetischen Fußsyndrom analysiert werden.

**Methode:** Die Kultivierung erfolgte unter optimalen aeroben und anaeroben Bedingungen. Die Identifizierung der bakteriellen Isolate wurde mit mikrobiologischen Standardmethoden vorgenommen. Die Testung der Antibiotikaempfindlichkeit erfolgte gemäß den Richtlinien des Clinical und Laboratory Standards Instituts (CLSI).

**Ergebnisse: **Von 60 Proben diabetischer Fußulcera wurden 92 Bakterienstämme isoliert. Dominierende Aerobier waren *S. aureus *(28%), gefolgt von Vertretern der Enterobacteriaceae (24%) einschließlich *Escherichia coli* (15%), *Citrobacter *spp. (4%), *Enterobacter *spp*.* (43%) und Coagulase-negativen *Staphylococcus *spp. (17%), *Enterococcus *spp. (15%), *Pseudomonas aeroginosa* (7%) und *Acinetobacter *spp. (4%). In den anaeroben Kulturen war in 4% der Ulcera *Bacteroides fragilis* nachweisbar, jedoch in keinem Fall *Clostridium *spp*.* Alle Gram-positiven Isolate waren gegen Linezolid empfindlich; alle Vertreter der Enterobacteriaceae waren gegenüber Imipenem empfindlich.

**Schlussfolgerung:** Die meisten Infektionen bei den diabetischen Fußulcera waren durch eine Mischflora mit Dominanz von *S. aureus* und* B. fragilis* gekennzeichnet. Die Ulcera können daher in der Initialtherapie sinnvollerweise eine kombinierte antimikrobielle Therapie erfordern.

## Introduction

Diabetes is a group of metabolic syndromes and falls into two groups. One group is type 1 diabetes, which accounts for only 5–10% of individuals with diabetes. Another group is type 2 diabetes, which accounts for 90–95% of individuals with this form of diabetes [1]. The International Diabetes Federation has anticipated that the number of persons with diabetes will increase from 240 million in 2007 to 380 million in 2025 [2]. People with diabetes, due to impaired micro vascular circulation, neuropathy, anatomical alterations, and impaired immune capacity are at higher risk than no diabetics for developing foot wounds infection [3]. Nephropathy leading to renal failure; retinopathy with loss of vision; peripheral neuropathy with risk of foot ulcers and amputations are long-term complications of diabetes [1]. In these patients ischemia, neuropathy, and infection integrates to produce tissue necrosis and ulcers [4]. Foot ulcers are most common in diabetic patients with prevalence about 25% and its long-term sequel, reason for direct medical costs of hundreds of millions of dollars annually, long periods of hospitalization and disability [5], [6], [7]. Diabetic foot ulcer frequently becomes infected (40%–80%) in these patients [6]. In fact, if an infection occurs in these ulcers, it can spread quite rapidly, leading to vast tissue destruction and subsequent amputation, because these ulcers are extremely susceptible to infections [8]. These patients frequently require minor or major amputations of the lower limbs (15–27%), and infection is the fundamental factor in more than 50% of cases [9]. The most important reason for non-traumatic lower extremity amputation is a diabetic foot ulcer and amputation is the most feared result in the life of the diabetic patient [10]. Rapid identification of factors contributing to this condition is appropriate for the successful resolution before it leads to amputation [10]. Several studies have shown, there is variation in the prevalence of common bacterial pathogens isolated, and most mild diabetic foot infections are mono microbial and are caused by aerobic Gram-positive cocci such as *S. aureus* and *Streptococcus *spp. The most severe infections are commonly poly microbial and caused by aerobic Gram-positive cocci (e.g., *S. aureus, Staphylococcus epidermidis *and* Enterococcus *spp*.*), Gram-negative bacilli (e.g., *Pseudomonas *spp*., Escherichia coli, Enterobacter *spp*.* and* Citrobacter *spp.) and anaerobes (e.g., *Bacteriodes *spp*., Peptostreptococcus *spp*., Fusobacterium *spp*. *and* Clostridium *spp.) [4], [11], [12], [13]. Initial treatment of diabetic foot infections is often empirical because reliable culture data is inaccessible [4]. Practical antibiotic therapy should be effective for these pathogens to prevent long term use of broad-spectrum antibiotics [14]. More recently, an increase in the prevalence of multi-drug resistant (MDR) organisms, mostly methicillin-resistant *S. aureus* (MRSA) and extended-spectrum β-lactamase (ESBL) producing Gram-negative bacteria, is menacing the result of anti-infectious treatment in the community and in hospitalized patients [9]. A bacteriological assessment of diabetic foot ulcer is essential to identify those agents that are involved in the development of these lesions. Knowledge of the bacteriology of diabetic foot infections is as well as significant in guiding antibiotic selection and appropriate definitive therapy that will help health care professionals to manage diabetic patients and prevent from subsequent amputation [10]. The aim of the study was the investigation of anaerobic and aerobic bacteria etiology and determination of antibiotic susceptibility pattern of isolated bacteria.

## Methods and material

### Collection of specimens

Between October 2013 and September 2014, 60 selected DFIs specimens were obtained from patients hospitalized in the Imam Reza hospital and Sina hospital. All patients were undergoing treatment with one or two of antibiotic drugs such as vancomycin, clindamycin, imipenem, ciprofloxacin, ceftriaxone and other cephalosporins.

All collected specimens were processed for detection of anaerobe and aerobe bacteria in the medical microbiology laboratory of medicine faculty. For sampling, diabetic foot infection site was first scrubbed with povidone-iodine and culture specimen were obtained by needle aspiration of material in depth of infected sites. First of all a drop of its content introduced to thioglycolate broth medium and then syringe was immediately sealed [15], [16]. Specimen were transported to laboratory within 20 min and it was generally inoculated at most within 1 h after collection.

### Microbial investigation

A Gram stain smear processed to cytology investigation and detection of bacterial presence in specimens. For the isolation of aerobic organisms, specimens were plated onto chocolate, sheep blood (5%), phenyl ethyl alcohol (PEA) and MacConkey agar plate. The plates were incubated at 37ºC under 10% CO_2_ and examined at 24 and 48 h. Pre-reduced vitamin K enriched brucella blood agar; kanamycin-vancomycin-laked blood agar (KVLB), bacteroides bile esculin (BBE) and phenyl ethyl alcohol (PEA) agar were inoculated for isolation of anaerobic organisms. The plate media were incubated under 80% N_2_, 10% CO_2_, 10% H_2_ and 0% O_2_ in an anaerobic jar by using Anoxomat (MART microbiology B.V. the Netherland) and these plates examined at 48, 72, and 96 h. The primary inoculated thioglycolate broth was incubated for 10 days and sub-cultured in 2 series of plates that was mentioned above in the same way. For enrichment and isolation of *Clostridium perfringens*, a drop of syringe specimen was introduced in cooked meat broth media and incubated at 45ºC for 4–6 h. Thereafter, one loop of this incubated media was sub-cultured in sheep blood agar plate and incubated under anaerobic condition and examined after 24 and 48 h [15], [17]. For identification of Gram-negative anaerobic bacteria, biochemical test such as reaction in Bile Esculin agar and MAST ID MID8 ANAEROB ID RING (MAST CO) was used [18].

### Phenotypic method for ESBL detection

#### Combined Disk Method (CDM)

Gram-negative bacilli isolates were inoculated onto Mueller Hinton agar and ceftazidime (30 µg) and ceftazidime/clavulanic acid (30 µg/10 µg) were placed at a center to center distance of at least 30 mm from each other. All plates were incubated at 37ºC for 18 hours and a >5 mm increase in inhibition zone of ceftazidime/clavulanic acid disks in comparison to its zone when tested alone and without clavulanic acid confirmed ESBL production [19].

#### Antibiotic susceptibility testing

For investigation of antibiotic susceptibility pattern in aerobic bacteria that were isolated from these infections, we performed antibiogramm test by Kirby-Bauer method (disk diffusion test) in Muller-Hinton agar and the guideline of CLSI was used [20].

Imipenem (10 µg), gentamicin (10 µg), amoxicillin-clavulanic acid (20/10 µg), ciprofloxacin (5 µg), cefoxitin (30 µg), tetracycline (30 µg), piperacillin-tazobactam (100/10 µg), ceftriaxone (30 µg), cefepime (30 µg), piperacillin (100 µg), ampicillin (10 µg) and colicin (10 µg) were tested for Gram-negative bacilli and erythromycin (15 µg), vancomycin (30 µg), clindamycin (2 µg), gentamicin (10 µg), cefoxitin (30 µg), oxacilin (1 µg), linezolid (30 µg), amoxicillin-clavulanic acid (20/10 µg), tetracycline (30 µg), ciprofloxacin (5 µg), ceftriaxone (30 µg), ampicillin (10 µg) and rifampicin (5 µg) were tested for Gram-positive bacteria isolated from these infections.

## Results

Forty-six (77%) male and fourteen (23%) female of diabetic cases with a mean age of 52 years and diabetic foot ulcers were studied during a determined period. The clinical characteristics of the patients with diabetic foot ulcers are shown in Table 1 (Tab. 1). The majority of the specimens came from hospitalized patients and aspiration was used for sample collection. In this study, we isolated 92 bacteria from 60 patients with an average of 1.7 organisms per lesion. Six patients (10%) had no bacterial growth in their media cultures. From 54 positive cultures, 22 cases (41%) were mono microbial and 32 patients (59%) had poly microbial infections, of which 26 were 2 types and 6 patients with 3 types of microorganisms. Aerobic Gram-positive cocci, represented 61% of the 92 bacterial isolates and Gram-negative facultative anaerobes bacilli and anaerobes represented 35% and 4% respectively. *Staphylococcus aureus* (28%) was the most commonly isolated from the patients with diabetic foot ulcers in our study, followed by coagulase-negative staphylococci (17%), *Enterococcus *spp. (15%). 

Enterobacteriaceae (24%) including *E. coli* (15%), *Citrobacter *spp. (4%), *Enterobacter *spp. (4%) was predominant Gram-negative facultative anaerobes, followed by *Pseudomonas aeruginosa* (7%), and *Acinetobacter *spp. (4%). Most frequently identified anaerobic bacteria from this study were *Bacteroides fragilis* (4%). 

**Figure 1:**
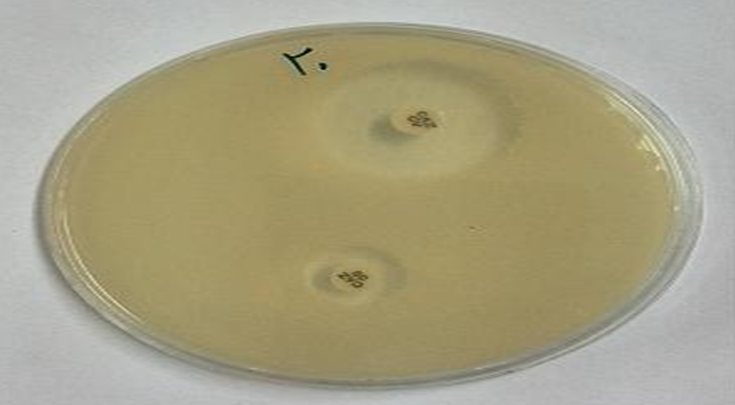
A positive combined disk test (CDT) using ceftazidime (CAZ 30 µg), ceftazidime/clavulanic acid (30 µg/10 µg) disks. A representative of ESBL producing isolates showing a >5 mm zone size enhancement in the CD test indicating inhibition of ESBL production.

Antimicrobial susceptibility pattern of these bacteria are shown in Table 2 (Tab. 2). Methicillin-resistant *S. aureus* (MRSA) was observed 39% of all *S. aureus* isolates and was susceptible to vancomycin and linezolid. All *Enterococcus *spp. were sensitive to linezolid, while 43% were resistant to vancomycin. Gram-negative bacilli were isolated as ESBL producers in 31%. These bacteria included *Acinetobacter *spp. (50%) followed by *E. coli* (36%), *P. aeruginosa* (33%) and *Enterobacter *spp. (25%) (Figure 1 (Fig. 1)). Imipenem, gentamicin, and cefepime were the most effective antimicrobial agents against isolated Gram-negative bacteria except *Acinetobacter *species.

## Discussion

According to some study, the rise of the prevalence of diabetic mellitus is associated with the increasing problem of infections among diabetic patients. Especially diabetic foot infection accounts for 20% of hospital admission [3]. Incidence of the population with diabetes in Iran is 8%, which is estimated to 3 million cases when Iranian population is aged 25–64 years, with the frequency of DFI estimated at 3% [21]. Diabetic foot infection are generally polymicrobial and both aerobic an anaerobic bacteria were isolated from these infections [4]. In our study, 60 specimens were collected from hospitalized diabetic patients suffering from diabetic foot infection.

Six patient’s specimens (10%) had no bacterial growth. In current study, monomicrobial etiology was found in 41% and polymicrobial 59% in positive culture specimens. Out of 54 positive cultures 92 bacteria strains were isolated that is, 1.5 bacteria per patient, which is similar to another study [11].

Similar to other studies carried by El-Tahawy [14] and Abdulrazad et al. [4] in this research *S. aureus* was a frequently common bacterial pathogen that was isolated from 26 patient specimens. In contrast, in another study carried by Ako-Nai et al. [10], *E. coli* was the frequently common bacterial pathogen while *P. aeruginosa* was reported as commonest pathogen by Shanker et al. [22]. Source of infection, use of antibiotic drug for treatment, sample collection method and different type of infection can influence of pathogens diversity in DFI [4], [10], [12], [14]. In addition of *S. aureus* we isolated other Gram-positive cocci such as coagulase-negative Staphylococci and *Enterococcus *spp. from patients which these findings are also corresponding other results obtained by some other researches [9], [14]. Previous use of anti-microbial drug may increase the prevalence of *Enterococcus *spp. in DFI [14]. Aerobic Gram-positive organisms were associated with mild and moderate forms of disease while in severe forms, infections were with significant increases in the number of Gram-negative organisms [4]. The situation of wounds got much worse and more severe with bacteria producing ESBL. In the present study ESBL producing Gram-negative bacteria was 31.3%. On confirmation of ESBL production by combined disk tests, highest prevalence of ESBL was observed in *Acinetobacter *spp*.* (50%) followed by *E. coli* (36%), *P. aeruginosa* (33%) and *Enterobacter *spp. (25%) which were consistent with the study carried out by Shobha et al. [23]. In our study 38.5% of *S. aureus* isolates were methicillin resistant that was determined by using the 30 µg cefoxitin disk [20]. This is in agreement with other results reported by Zubair et al. [13] and El-Tahawy [14]. This could be due to prolonged antibiotic therapy and administration of broad-spectrum antibiotics that may increase prevalence of antibiotic resistance organism like MRSA or vancomycin resistant *Enterococcus *spp. (VRE) in DFI.

In our research among Gram-negative pathogens *E. coli* was the most isolated organism which is in agreement with the results reported by Hadadi Azar et al. [24]. Similar to other studies all *E. coli* strains isolated in our study were imipenem sensitive, but different levels of resistance to other antibiotics were observed the same as previous reports [11], [24]. In fact, unnecessary and frequent use of antibiotic can result in selection of antibiotic resistant organisms. Such organisms could be transmitted from infected patient to patient with diabetic foot ulcers by the health center stuff [8].

In the current study, 32 (59%) of cases had polymicrobial infection (2 bacteria isolated from 22 patient and 3 bacteria from 6 patient) which was in accordance with another study carried by Zubair et al. [13] that reported polymicrobial infection in 62% of patient. We isolated *Bacteroides fragilis* from 4 patients with polymicrobial infection. Other researchers such as Shanker et al. [22] reported *Bacteroides fragilis* as frequently common anaerobic bacteria in DFI. All anaerobic bacteria isolated from polymicrobial ulcer were usually associated with facultative or aerobic bacteria [14]. Our anaerobic bacteria report was less than other studies because most of our study population did not have chronic infection, which is consistent with the previous study carried by Zubair et al. [13].

All *S. aureus* isolated were susceptible to vancomycin and linezolid and all of *Enterococcus *spp. susceptible to Linezolid. These antibiotics are highly effective against Gram-positive cocci isolated from this study and these antibiotics seem to be appropriate for empirical treatment of DFIs. Emergence of bacterial strains resistant to clindamycin and ciprofloxacin decreases effectiveness of these drugs for empirical therapy. The findings of this research indicated that imipenem is highly effective against Gram-negative bacteria; therefore this antibiotic could be suitable for use in empirical therapy. The results obtained show that diabetic foot infections are often poly microbial and in most cases associated with *S. aureus, E. coli *and* Enterococcus *spp*. *The diversity of microbial profile and resistance to antibiotic drugs in this study emphasize the need to obtain culture specimens from infected ulcers for microbial assessment and antibiotic susceptibility testing.

The present study suggests that for correct management of the DFI knowledge of the susceptibility of antimicrobial drug for choice of appropriate antibiotics with maximum effectiveness is necessary.

## Notes

### Conflict of interests

The authors declare that they have no conflict of interests.

## Acknowledgements

This research was supported by a grant from Infectious and Tropical Disease Research Center of Tabriz University of Medical Sciences (TUMS) and the manuscript was written based on a dataset of MSc thesis of Naser Alizadeh registered at Tabriz University of Medical Sciences. The authors would like to thank the staff of Imam Reza and Sina infectious disease wards and microbiology department for their help. The Ethic Commission of Tabriz University of Medical Sciences approved this study (Number: 5/4/589 -23 Mar.2014).

## Figures and Tables

**Table 1:**
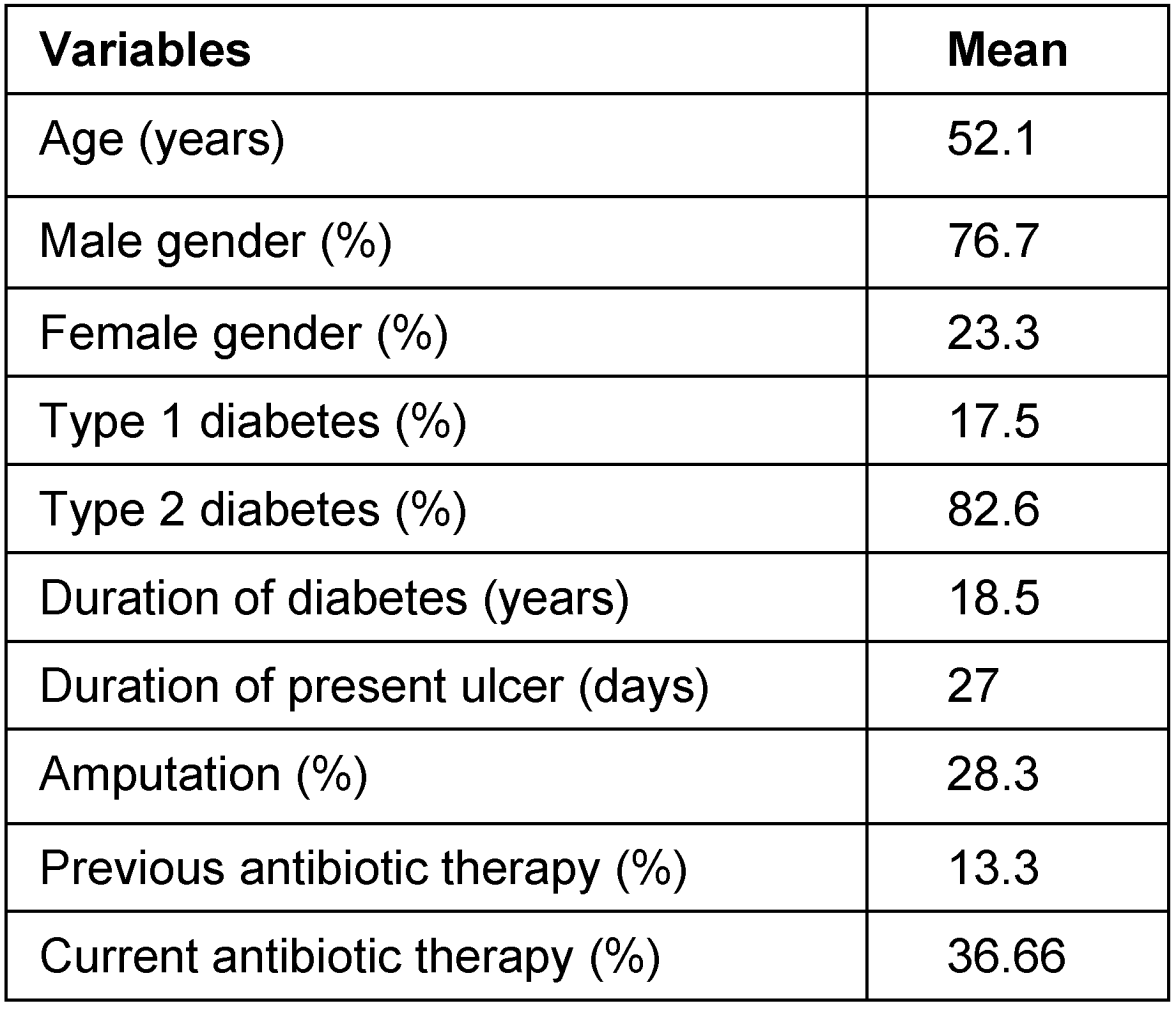
Clinical characteristics of the patients with diabetic foot ulcers

**Table 2:**
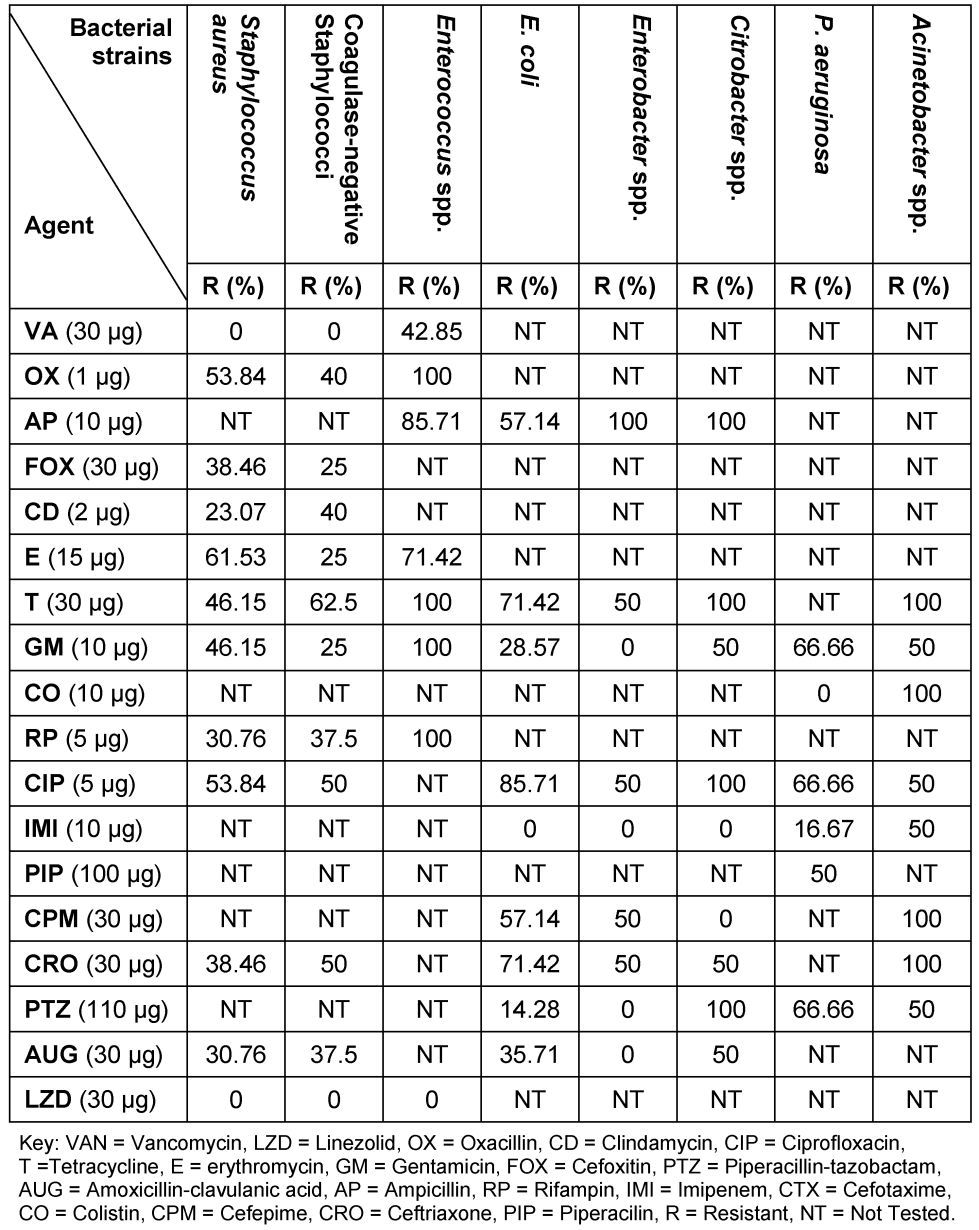
Antimicrobial susceptibility pattern of bacterial isolated
